# Pembrolizumab-combination therapy for previously untreated metastatic nonsquamous NSCLC: Real-world outcomes at US oncology practices

**DOI:** 10.3389/fonc.2022.999343

**Published:** 2022-10-17

**Authors:** Stephen V. Liu, Xiaohan Hu, Yeran Li, Bin Zhao, Thomas Burke, Vamsidhar Velcheti

**Affiliations:** ^1^ Lombardi Comprehensive Cancer Center, Georgetown University, Washington, DC, United States; ^2^ Center for Observational and Real-world Evidence, Merck & Co., Inc., Rahway, NJ, United States; ^3^ Clinical Research, Merck & Co., Inc., Rahway, NJ, United States; ^4^ Perlmutter Cancer Center, New York University (NYU) Langone Health, New York, NY, United States

**Keywords:** observational study, pembrolizumab, chemotherapy, overall survival, manual chart review, real-world progression-free survival, real-world response rate

## Abstract

**Objectives:**

The availability of immunotherapies has expanded the options for treating metastatic NSCLC, but information is needed regarding outcomes of immunotherapy for patients treated outside of clinical trials. The aim of this retrospective study was to evaluate the outcomes of therapy with first-line pembrolizumab plus pemetrexed and carboplatin (pembrolizumab-combination) for patients with metastatic nonsquamous NSCLC in the real-world setting of oncology clinics in the United States (US).

**Methods:**

Using deidentified, longitudinal patient records from a nationwide, electronic health record-derived US database, we identified patients with metastatic nonsquamous NSCLC, without *EGFR*/*ALK/ROS1* genomic alterations, who had received no previous systemic anticancer therapy. Eligible patients had an Eastern Cooperative Oncology Group (ECOG) performance status of 0 or 1 and initiated first-line pembrolizumab-combination therapy from 11-May-2017 to 31-January-2019; data cutoff was 31-August-2020. Patients treated in a clinical trial were excluded. Manual chart review supplemented technology-enabled abstraction to identify disease progression and tumor response. Time-to-event endpoints from initiation of pembrolizumab-combination therapy were determined using Kaplan-Meier.

**Results:**

Of 377 patients with metastatic nonsquamous NSCLC, 105 (28%), 104 (28%), and 103 (27%) had programmed death-ligand 1 (PD-L1) expression ≥50%, 1–49%, and <1%, respectively; PD-L1 expression was not documented for 65 patients (17%). Median age was 66 years, and 227 patients (60%) were men. Median follow-up time from first-line therapy initiation to data cutoff was 31.2 months (range, 19.0-39.6 months). Median pembrolizumab real-world time on treatment (rwToT) was 5.8 months (95% CI, 5.0-6.7); 12- and 24-month on-treatment rates for pembrolizumab were 28.0% and 14.9%, respectively. Median overall survival (OS) was 17.2 months (95% CI, 13.6-19.9). For patients in PD-L1 expression ≥50%, 1-49%, <1%, and unknown cohorts, the 12-month survival rates were 66.0%, 58.5%, 54.5%, and 58.3%, respectively, and 24-month survival rates were 43.1%, 37.2%, 35.6%, and 42.0%, respectively. Median real-world progression-free survival was 6.2 months (95% CI, 5.5-7.1); and the real-world response rate was 39.3%, with median duration of response of 13.1 months (95% CI, 10.5-16.8).

**Conclusions:**

These findings demonstrate the benefits of first-line pembrolizumab-combination therapy for patients with *EGFR/ALK*-wild-type, metastatic nonsquamous NSCLC and good performance status who are treated at US community oncology clinics.

## Introduction

Comparative clinical trials are designed to minimize confounding factors and maximize internal validity by selecting patients according to strict eligibility criteria and then providing therapy under idealized conditions with frequent follow-up. The results of these trials provide important efficacy and safety information used by medical oncologists to select the optimal course(s) of systemic anticancer therapy for their patients. However, outside of clinical trials, oncologists face the challenges of treating a heterogeneous population of patients, often with characteristics not represented in clinical trials, and of working under time, financial, and other practical constraints. Thus, the importance of understanding clinical outcomes of anticancer therapies for so-called “real-world” patients is increasingly noted and incorporated into regulatory frameworks (1–3).

For patients with metastatic non-small cell lung cancer (NSCLC), the recent availability of immunotherapies, including immune checkpoint inhibitors (ICI) of programmed death 1 (PD-1) and PD-ligand 1 (PD-L1), has expanded the options for therapy (4, 5). The combination of pembrolizumab with pemetrexed and platinum was first approved for patients with previously untreated metastatic nonsquamous NSCLC on 10 May 2017 in the United States (US) under the Food and Drug Administration (FDA) accelerated approval process, with conversion to full approval on 20 August 2018 (6). Pembrolizumab plus pemetrexed and platinum chemotherapy is currently the category 1, preferred regimen in US National Comprehensive Cancer Network guidelines for patients with advanced or metastatic nonsquamous NSCLC, no targetable genomic aberrations, and good performance status (Eastern Cooperative Oncology Group performance status [ECOG PS] of 0 or 1), independent of tumor PD-L1 expression (7). After four cycles of combination therapy, pembrolizumab can be continued for up to 24 months and maintenance pemetrexed is administered until disease progression or unacceptable toxicity.

The efficacy of this combination regimen is supported by KEYNOTE-189 and -021 clinical trial results demonstrating superior overall survival (OS) for patients with advanced or metastatic nonsquamous NSCLC and no *EGFR* or *ALK* genomic aberrations who were treated with first-line pembrolizumab plus pemetrexed-platinum as compared with placebo plus pemetrexed-platinum (8–12). To date, however, a limited number of published studies have assessed survival outcomes of real-world patient populations treated with first-line immunotherapy-chemotherapy combinations in the US (13–19). An earlier study reported the effectiveness of first-line pembrolizumab plus pemetrexed-carboplatin for 283 patients with metastatic nonsquamous NSCLC treated at US oncology clinics: median OS was 16.5 months, and the 12-month survival rate was 59.5% (17). Study follow-up ended in August 2019. The aim of the present retrospective study was to update those findings with longer follow-up of a larger patient population, using the same database derived from electronic health records (EHRs).

## Methods

### Data source and patients

The data for this retrospective observational study were drawn from the Flatiron Health database, containing deidentified, longitudinal EHR-derived data of patients with cancer treated at oncology clinics throughout the US (20, 21). The database, used frequently for research purposes, comprises patient-level structured and unstructured data, curated *via* technology-enabled abstraction, as previously described (22–24). At the time of the present study, EHR data originated from approximately 280 cancer clinics (~800 sites of care).

We studied adult patients (≥18 years old) with pathologically confirmed metastatic nonsquamous NSCLC and documented wild-type *EGFR* and *ALK*, plus no known *ROS1* rearrangement. Eligible patients had an ECOG PS of 0 or 1 and initiated first-line therapy with pembrolizumab plus pemetrexed and carboplatin (pembrolizumab-combination) during the period from 11 May 2017 to 31 January 2019. Data cutoff was 31 August 2020 (minimum potential study follow-up thus 19 months). Patients who received first-line therapy in a clinical trial, and those with no recorded activity in the database within 90 days (inclusive) of the metastatic NSCLC diagnosis, were excluded. While some patients were likely included in our earlier study (17), the rules protecting against patient reidentification prevented us from determining how many and who they were.

The study was conducted according to the guidelines of the Declaration of Helsinki, and ethical approval of the study protocol was obtained from the WCG Institutional Review Board before study conduct, including a waiver of informed consent for working with deidentified data. Safeguards were in place to maintain data deidentification. Flatiron Health, Inc. did not participate in the analysis of the data.

### Assessments

We determined OS, real-world time on treatment (rwToT) with pembrolizumab, and rwToT with pemetrexed from the time of initiating first-line pembrolizumab-combination therapy. The OS endpoint was determined using the validated Flatiron Health mortality 2.0 endpoint (25, 26), with date of death set to the 15th of the month to maintain data deidentification; patients with no recorded death were censored at the last contact date or at data cutoff, whichever occurred first. Pembrolizumab rwToT was defined as the time from first to last recorded dose of first-line pembrolizumab, with discontinuation pegged at the last administration date if patients initiated second-line therapy or had a gap in therapy of ≥120 days; patients meeting none of these discontinuation criteria were censored at their last pembrolizumab administration date (27). Pemetrexed rwToT was calculated similarly. Subsequent lines of therapy were identified using the Flatiron Health oncologist-defined business rules, with mapping of medication administrations and medication orders to lines of therapy (20).

Trained chart abstractors used manual chart review to determine additional clinical outcomes. Real-world progression (rwP) was identified as each episode (>14 days after first-line initiation) when the treating clinician concluded that there was growth or worsening of NSCLC (24). Real-world progression-free survival (rwPFS) was then determined as the time from first-line therapy initiation to the first documented rwP event or death from any cause, whichever occurred first, with censoring at the date of the last clinical note before patients initiated a second-line therapy and date of the last clinical note for those with no subsequent therapy.

The real-world response (rwR) was determined using clinicians’ assessment of change in disease burden after radiographic imaging, limited to assessments made ≥30 days after first-line initiation, and mapped to complete response (CR), partial response (PR), stable disease, progressive disease (PD), or other (indeterminate, pseudoprogression, not documented), as previously described (28). The real-world response rate (rwRR) was calculated for patients who had at least one CR or PR assessment followed by a subsequent assessment of PR, CR, or stable disease during first-line therapy. The duration of response (rwDOR) was defined as the time from first documented evidence of CR or PR until disease progression or death; and patients with no record of disease progression or death were censored at the date of their last rwR assessment.

In addition, the reasons for first-line pembrolizumab discontinuation were captured from physician’s notes or because of gap in therapy of >60 days and classified by chart abstractors according to predefined categories (see Results section for categories as reported).

### Statistical analyses

Summary statistics were used to describe, overall and by PD-L1 expression, baseline demographic and clinical patient characteristics, as well as the number of pembrolizumab cycles, reasons for first-line pembrolizumab discontinuation, and systemic anticancer therapies administered after first-line therapy. The Charlson comorbidity index (CCI) score was derived from listed comorbidities as described by Khan et al. (29).

The Kaplan-Meier method was used to analyze time-to-event endpoints, overall and by PD-L1 expression, including rwToT, OS, rwPFS, and rwDoR. A sensitivity analysis of rwPFS was conducted that censored patients with rwP events labeled as pseudoprogression, defined as an increase in tumor size that the clinician recorded as possibly an effect of immunotherapy. Outcome results by PD-L1 expression were not compared statistically.

Statistical analyses were conducted using SAS software, version 9.4 (SAS Institute, Cary, NC).

## Results

### Patients and therapy

**Figure 1:**
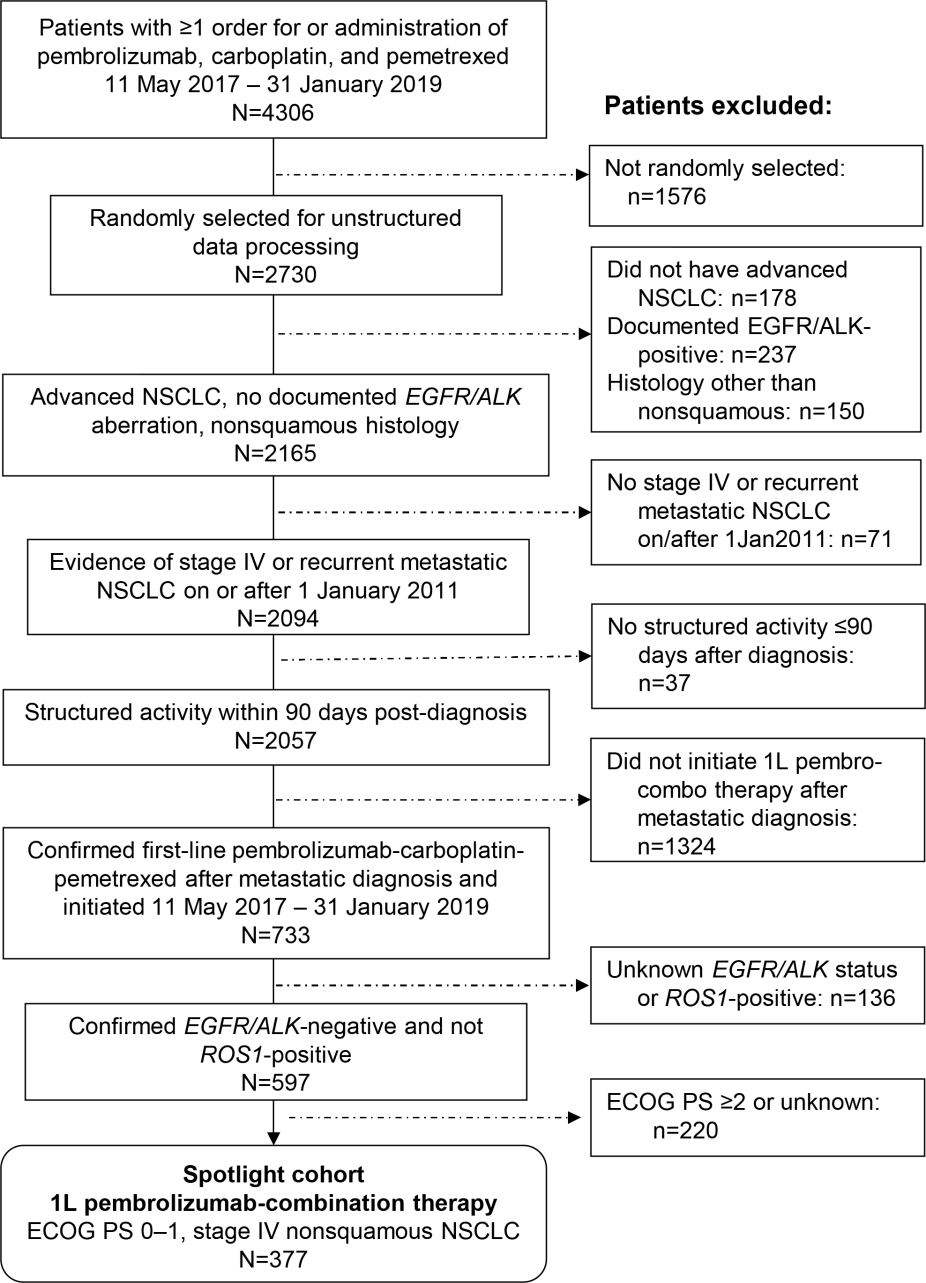
Patient selection from the Flatiron Health Database. 1L, first-line; ECOG PS, Eastern Cooperative Oncology Group performance status.

Of 377 patients with metastatic nonsquamous NSCLC (**Figure 1**), 105 (28%), 104 (28%), and 103 (27%) had PD-L1 expression of ≥50%, 1–49%, and <1%, respectively; PD-L1 expression was not documented for 65 patients (17%). The first diagnosis of NSCLC was made at stage IIIB or IV for most patients (303; 80%), whereas the other patients either experienced recurrence or progression from an earlier initial stage (29; 8%) or had an unknown initial stage (45; 12%). The median age overall was 66 years, and 227 patients (60%) were men (**Table 1**). Most patients were treated at community oncology clinics; only 9 patients (2%) were treated at academic centers.

**Table 1:** Patient characteristics at initiation of first-line pembrolizumab-combination therapy.

		PD-L1 expression level			
Characteristic	All patients N = 377	≥50% n = 105	1–49% n = 104	<1% n = 103	Unknown n = 65
Sex, male	227 (60.2)	67 (63.8)	56 (53.8)	67 (65.0)	37 (56.9)
Age, median (range)	66 (29–83)	67 (46–83)	67 (46–83)	66 (34–82)	66 (29–82)
Age group					
<65 years	155 (41.1)	41 (39.0)	42 (40.4)	45 (43.7)	27 (41.5)
65–74 years	143 (37.9)	40 (38.1)	38 (36.5)	40 (38.8)	25 (38.5)
≥75 years	79 (21.0)	24 (22.9)	24 (23.1)	18 (17.5)	13 (20.0)
Race^a^					
White	256 (76.4)	71 (75.5)	74 (78.7)	64 (71.9)	47 (81.0)
Black or African American	40 (11.9)	10 (10.6)	11 (11.7)	14 (15.7)	5 (8.6)
Asian	6 (1.8)	2 (2.1)	1 (1.1)	2 (2.2)	1 (1.7)
Other	33 (9.9)	11 (11.7)	8 (8.5)	9 (10.1)	5 (8.6)
Unknown	42	11	10	14	7
Smoking status					
History of smoking	345 (91.5)	97 (92.4)	96 (92.3)	94 (91.3)	58 (89.2)
No history of smoking	32 (8.5)	8 (7.6)	8 (7.7)	9 (8.7)	7 (10.8)
*BRAF* mutation status^a,b^					
*BRAF* mutant	12 (4.2)	4 (5.3)	3 (3.9)	2 (2.5)	3 (5.7)
Wild-type	272 (95.8)	71 (94.7)	73 (96.1)	78 (97.5)	50 (94.3)
Unknown/missing	93	30	28	23	12
*KRAS* mutation status^a,b^					
*KRAS* mutant	109 (42.1)	43 (61.4)	27 (42.9)	19 (25.7)	20 (38.5)
*KRAS* wild-type	150 (57.9)	27 (38.6)	36 (57.1)	55 (74.3)	32 (61.5)
Unknown/missing	118	35	41	29	13
Brain metastasis, yes	51 (13.5)	15 (14.3)	15 (14.4)	16 (15.5)	5 (7.7)
ECOG performance status^c^					
0	164 (43.5)	48 (45.7)	45 (43.3)	41 (39.8)	30 (46.2)
1	213 (56.5)	57 (54.3)	59 (56.7)	62 (60.2)	35 (53.8)
Charlson comorbidity index					
Mean (SD)	3.6 (3.3)	3.6 (3.4)	3.5 (3.2)	3.9 (3.3)	3.5 (3.3)
Median (range)	3 (0–13)	2 (0–13)	2.5 (0–10)	6 (0–11)	2 (0–10)
IHC clone for PD-L1 determination					
22C3^d^	267 (70.8)	89 (84.8)	84 (80.8)	94 (91.3)	0
SP263	12 (3.2)	5 (4.8)	4 (3.8)	3 (2.9)	0
Other	7 (1.9)	2 (1.9)	3 (2.9)	2 (1.9)	0
Unknown/missing/not documented	91 (24.1)	9 (8.6)	13 (12.5)	4 (3.9)	65 (100)

Data are n (%) unless otherwise noted. Percentages may not add up to 100 because of rounding.

a Percentages for race, *BRAF* status, and *KRAS* status represent the percentages of patients with available data.

b Positive biomarker results at any time (“ever positive”) were included.

c ECOG performance status recorded during the time period from metastatic NSCLC diagnosis to 30 days after first-line therapy initiation.

d Of the 22C3 IHC assays, 255/267 (96%) used the PD-L1 IHC 22C3 pharmDx, pembrolizumab companion diagnostic assay.

ECOG, Eastern Cooperative Oncology Group; IHC, immunohistochemistry; PD-L1, programmed death-ligand 1.

All patients had *EGFR* and *ALK* wild-type nonsquamous NSCLC; and 342 (91%) had known *ROS1* wild-type NSCLC (*ROS1* rearrangement status was unknown for 35 patients). Of 259 patients with known *KRAS* mutation status, 109 (42%) had *KRAS*-mutant NSCLC, including 61%, 43%, and 26% in PD-L1 ≥50%, 1–49%, and <1% cohorts, respectively (**Table 1**). The pembrolizumab companion diagnostic assay (PD-L1 IHC 22C3 pharmDx, Dako, Agilent, Carpinteria, CA) was used most commonly to determine PD-L1 expression, including for 255 of the 286 patients (89%) with known immunohistochemistry assay type.

The median study follow-up time from first-line therapy initiation to data cutoff (31 August 2020) was 31.2 months (range, 19.0–39.6 months). The median patient follow-up time from first-line therapy initiation to death or data cutoff, whichever occurred first, was 18.9 months (range, 1 day to 39.5 months).

**Figure 2:**
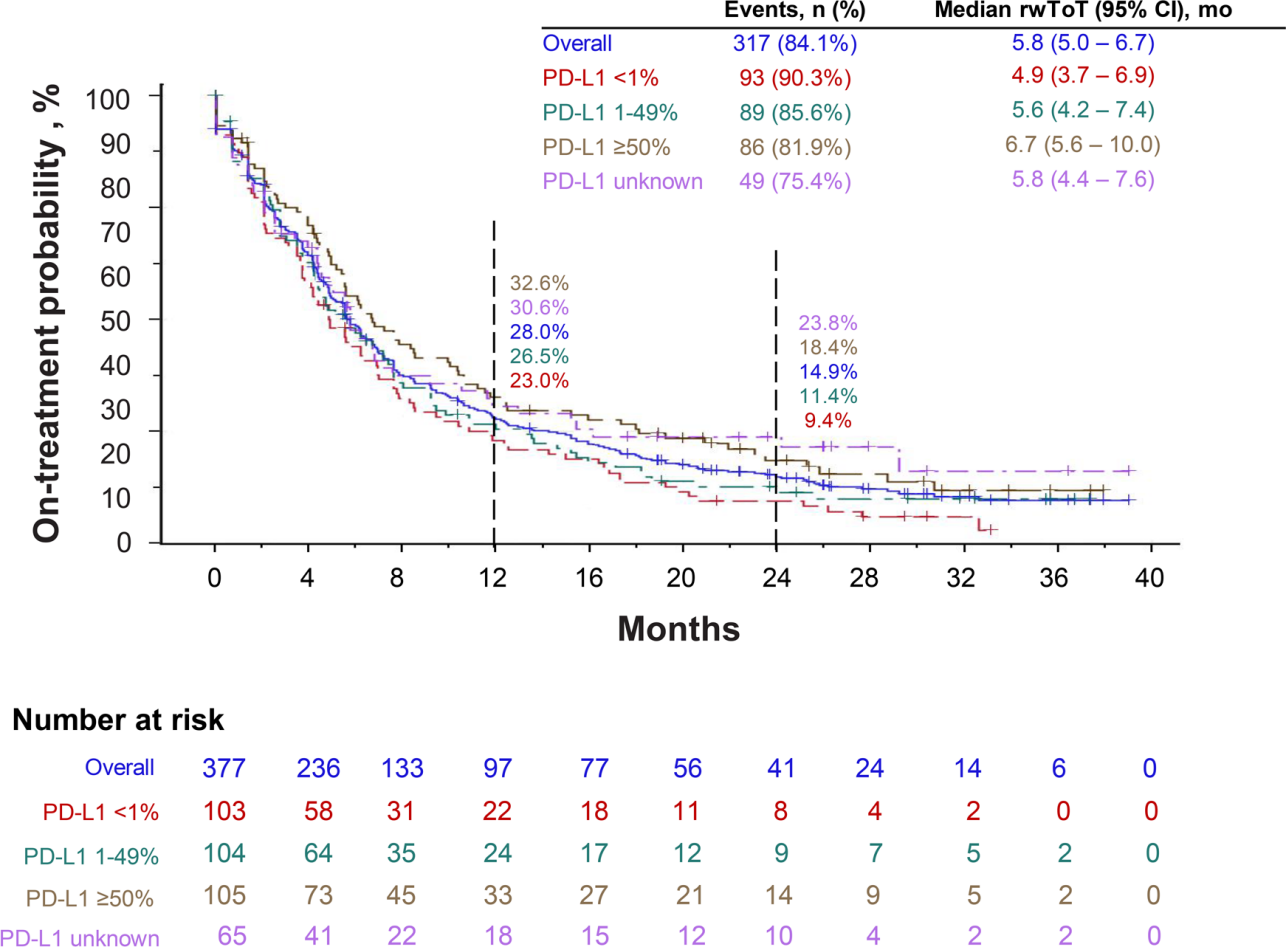
Real-world time on treatment (rwToT) with first-line pembrolizumab, when administered in combination with pemetrexed and carboplatin.

Overall, the median pembrolizumab rwToT was 5.8 months (95% CI, 5.0–6.7), and the Kaplan-Meier estimated on-treatment rates for pembrolizumab were 28.0% (95% CI, 23.4–32.7) at 12 months and 14.9% (95% CI, 11.3–18.9) at 24 months (**Figure 2**). The 12-month on-treatment rates were 32.6%, 26.5%, 23.0%, and 30.6% in PD-L1 ≥50%, 1–49%, <1%, and unknown cohorts, respectively, while the 24-month on-treatment rates were 18.4%, 11.4%, 9.4%, and 23.8%, respectively. Patients received a median of 9 pembrolizumab cycles overall (range 1–64 cycles; **Table 2**). At the time of data cutoff, 272 patients (72%) had discontinued pembrolizumab therapy, most commonly because of disease progression (145/377; 38%; **Table 3**).

**Table 2:** Systemic anticancer therapy lines and regimens.

		PD-L1 expression level			
Therapy line and regimen	All patients N = 377	≥50% n = 105	1–49% n = 104	<1% n = 103	Unknown n = 65
**First-line pembrolizumab-chemotherapy**					
Pembrolizumab cycles, median (range)	9 (1–64)	10 (1–55)	8 (1–54)	7 (1–45)	9 (1–64)
Received ≥6 cycles, n (%)	253 (67.1)	75 (71.4)	71 (68.3)	65 (63.1)	42 (64.6)
Received ≥35 cycles, n (%)	40 (10.6)	14 (13.3)	9 (8.7)	8 (7.8)	9 (13.8)
**Second-line regimen, n (%)**	**147 (39.0)**	**42 (40.0)**	**45 (43.3)**	**43 (41.7)**	**17 (26.2)**
Anti-PD-1/PD-L1-based therapy	35 (23.8)	8 (19.0)	16 (35.6)	5 (11.6)	6 (35.3)
Anti-VEGF-based therapy	43 (29.3)	13 (31.0)	6 (13.3)	20 (46.5)	4 (23.5)
Platinum-based chemotherapy combination	16 (10.9)	5 (11.9)	6 (13.3)	3 (7.0)	2 (11.8)
Nonplatinum-based chemo combination	7 (4.8)	2 (4.8)	1 (2.2)	4 (9.3)	0
Single agent chemotherapy	28 (19.0)	8 (19.0)	9 (20.0)	8 (18.6)	3 (17.6)
Other therapy	18 (12.2)	6 (14.3)	7 (15.6)	3 (7.0)	2 (11.8)
**Third-line regimen, n (%)**	**65 (17.2)**	**16 (15.2)**	**20 (19.2)**	**21 (20.4)**	**8 (12.3)**
Anti-PD-1/PD-L1-based therapy	10 (15.4)	4 (25.0)	3 (15.0)	2 (9.5)	1 (12.5)
Anti-VEGF-based therapy	21 (32.3)	3 (18.8)	9 (45.0)	6 (28.6)	3 (37.5)
Platinum-based chemotherapy combination	3 (4.6)	0	1 (5.0)	1 (4.8)	1 (12.5)
Nonplatinum-based chemo combination	4 (6.2)	0	0	3 (14.3)	1 (12.5)
Single agent chemotherapy	23 (35.4)	6 (37.5)	7 (35.0)	8 (38.1)	2 (25.0)
Other therapy	4 (6.2)	3 (18.8)	0	1 (4.8)	0
**Fourth-line regimen, n (%)**	**26 (6.9)**	**8 (7.6)**	**8 (7.7)**	**9 (8.7)**	**1 (1.5)**
Anti-PD-1/PD-L1-based therapy	2 (7.7)	1 (12.5)	0	1 (11.1)	0
Anti-VEGF-based therapy	5 (19.2)	1 (12.5)	2 (25.0)	1 (11.1)	1 (100)
Platinum-based chemotherapy combination	1 (3.8)	0	0	1 (11.1)	0
Nonplatinum-based chemo combination	4 (15.4)	2 (25.0)	1 (12.5)	1 (11.1)	0
Single agent chemotherapy	11 (42.3)	3 (37.5)	4 (50.0)	4 (44.4)	0
Other therapy	3 (11.5)	1 (12.5)	1 (12.5)	1 (11.1)	0
**Fifth-line regimen, n (%)**	**8 (2.1)**	**1 (1.0)**	**2 (1.9)**	**4 (3.9)**	**1 (1.5)**
Anti-VEGF-based therapy	2 (25.0)	0	1 (50.0)	0	1 (100)
Platinum-based chemotherapy combination	1 (12.5)	0	1 (50.0)	0	0
Single agent chemotherapy	4 (50.0)	1 (100)	0	3 (75.0)	0
Other therapy	1 (12.5)	0	0	1 (25.0)	0
**Sixth-line regimen, n (%)**	**2 (0.5)**	**0**	**2 (1.9)**	**0**	**0**
Anti-PD-1/PD-L1-based therapy	1 (50.0)	0	1 (50.0)	0	0
Single agent chemotherapy	1 (50.0)	0	1 (50.0)	0	0

Drug regimens are shown as percentage of the relevant treatment line. Percentages may not total 100 because of rounding. For each line of therapy, mutually exclusive regimen classes were assigned in hierarchical order as follows: anti-PD-1/PD-L1-based therapy > anti-VEGF-based therapy > platinum-based chemotherapy combinations > nonplatinum-based chemotherapy combinations > single agent chemotherapy > other therapy.

Chemo, chemotherapy; PD-1, programmed death 1; PD-L1, PD-ligand 1; VEGF, vascular endothelial growth factor.Bold numbers (%) of patients receiving each subsequent line of therapy.

**Table 3:** Reasons for first-line pembrolizumab discontinuation.

		PD-L1 expression level			
	All patients N = 377	≥50% n = 105	–49% n = 104	<1% n = 103	Unknown n = 65
Discontinued, n (%)	272 (72.1)	76 (72.4)	76 (73.1)	81 (78.6)	39 (60.0)
Reasons for discontinuation, n (%)^a^					
Progression	145 (53.3)	33 (43.4)	46 (60.5)	46 (56.8)	20 (51.3)
Adverse events related to therapy	43 (15.8)	16 (21.1)	10 (13.2)	12 (14.8)	5 (12.8)
Disease-related symptoms not due to therapy	28 (10.3)	5 (6.6)	7 (9.2)	9 (11.1)	7 (17.9)
Patient request	13 (4.8)	5 (6.6)	4 (5.3)	3 (3.7)	1 (2.6)
Completed treatment	7 (2.6)	4 (5.3)	2 (2.6)	0	1 (2.6)
No evidence of disease	5 (1.8)	3 (3.9)	0	1 (1.2)	1 (2.6)
Financial	3 (1.1)	1 (1.3)	1 (1.3)	1 (1.2)	0
Other^b^	31 (11.4)	10 (13.2)	7 (9.2)	10 (12.3)	4 (10.3)
Unknown	2 (0.7)	1 (1.3)	0	1 (1.2)	0

Data are n (%) unless otherwise noted. Percentages may not add up to 100 because of rounding.

a Patients could have more than one reason for discontinuation.

b For patients with ongoing treatment until the time of death, the reason recorded was “Other” to comply with data deidentification requirements.

The median pemetrexed rwToT was 2.7 months (95% CI, 2.4–3.5) overall and 2.4 months (95% CI, 2.1–3.5), 3.5 months (95% CI, 2.2–4.4), 3.0 months (95% CI, 2.2–3.6) and 2.5 months (95% CI, 2.1–3.9) in PD-L1 ≥50%, 1-49%, <1% and unknown cohorts.

A total of 147 patients (39%) including 42 (40%), 45 (43%), 43 (42%), and 17 (26%) in PD-L1 ≥50%, 1–49%, <1%, and unknown cohorts, respectively, received one or more subsequent systemic anticancer regimens, as summarized in **Table 2** and detailed in **Supplementary Table 1**.

Forty-three patients were administered a PD-1/PD-L1 ICI also as second-line therapy (36 patients) and/or third-line therapy (10 patients). In second-line, the median ICI rwToT was 2.8 months (95% CI, 1.5–5.1) with ICI on-treatment rate of 27.4% at 12 months; and in third-line, the median ICI rwToT was 4.3 months (95% CI, 1.4 to not assessable) with on-treatment rate of 46.9% at 6 months (**Supplementary Table 2**). Their reasons for discontinuation of first-line pembrolizumab were similar to those of all patients, again with disease progression being the most common reason (**Supplementary Table 3**).

### Clinical outcomes

**Figure 3:**
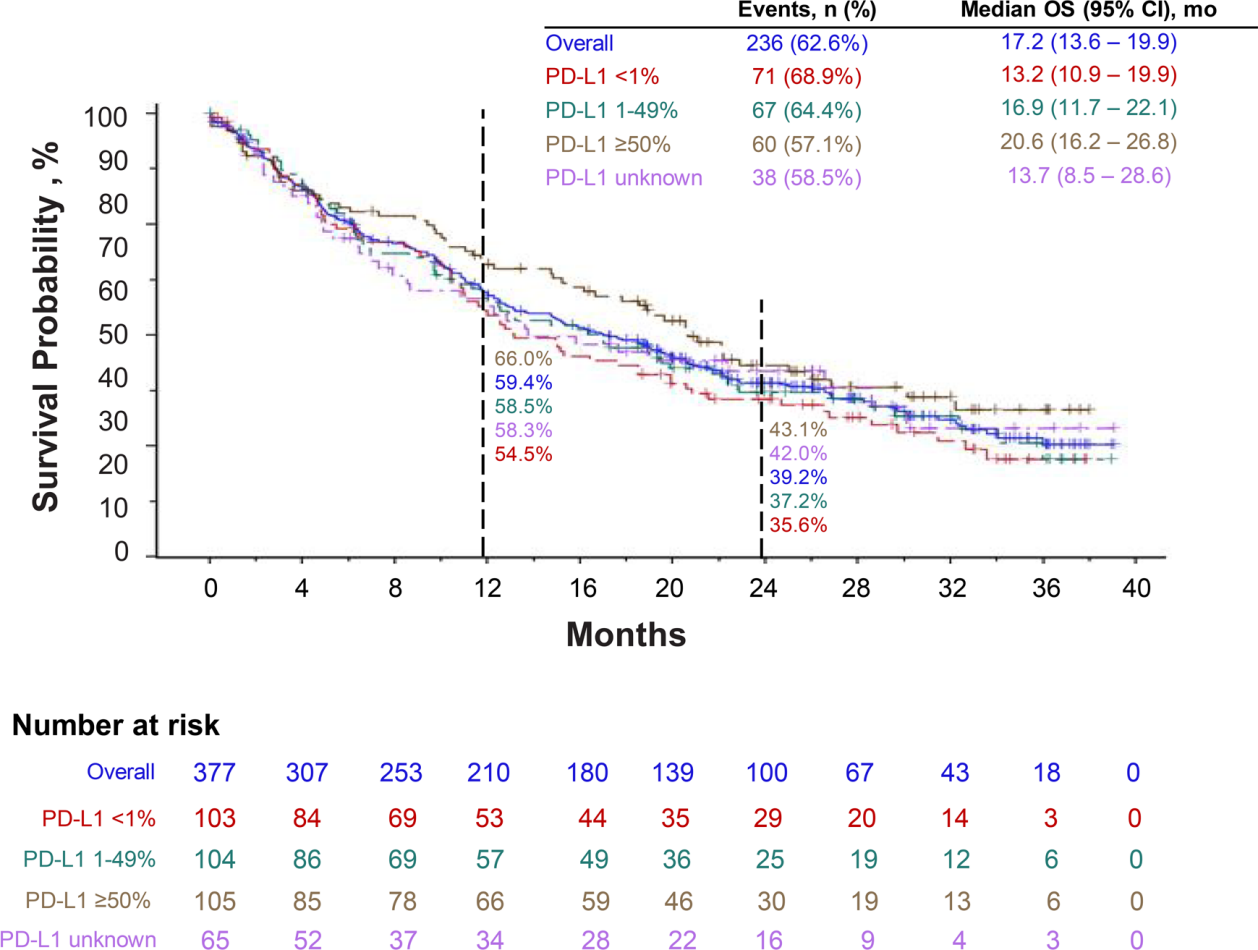
Overall survival with pembrolizumab plus pemetrexed and carboplatin.

**Figure 4:**
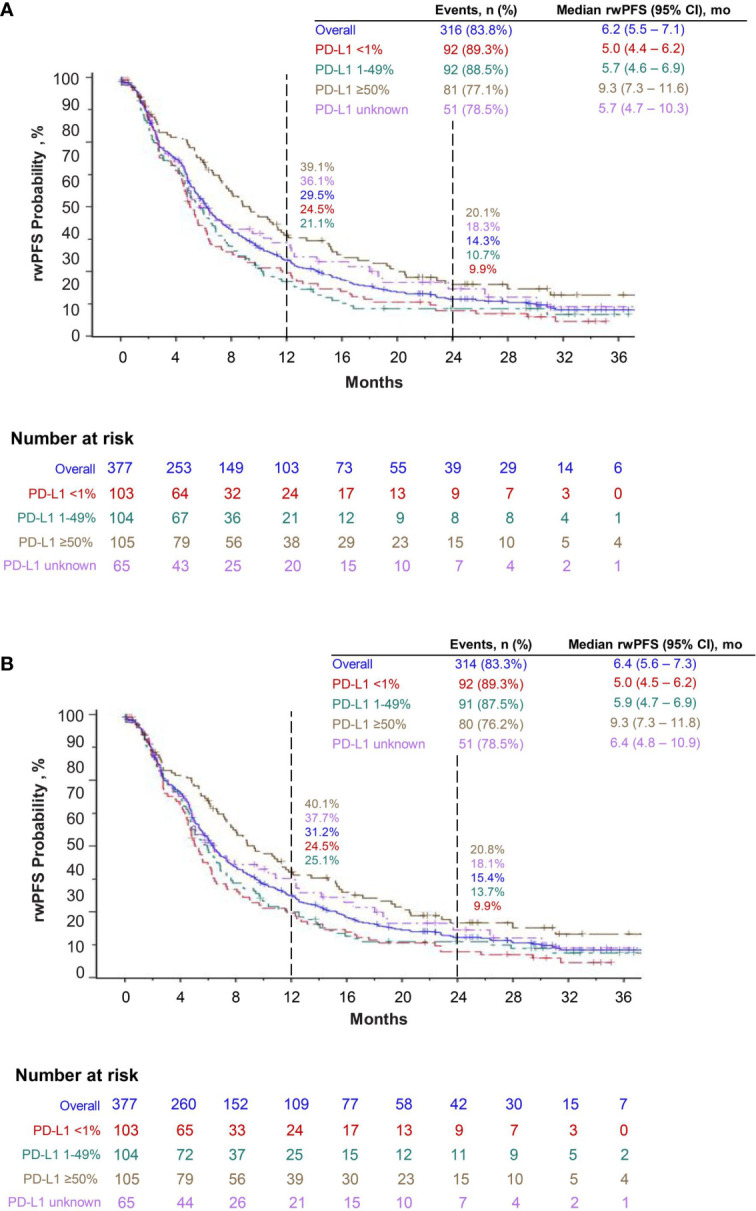
Real-world progression-free survival (rwPFS) with pembrolizumab plus pemetrexed and carboplatin **(A)** and sensitivity analysis of real-world progression-free survival, censoring pseudoprogression **(B)**.

At the time of data cutoff, median OS was 17.2 months (95% CI, 13.6–19.9). Overall, the estimated 12-month survival rate was 59.4%, and the 24-month survival rate was 39.2%. For patients in PD-L1 ≥50%, 1–49%, <1%, and unknown cohorts, the 12-month survival rates were 66.0%, 58.5%, 54.5%, and 58.3%, respectively, and the 24-month survival rates were 43.1%, 37.2%, 35.6%, and 42.0%, respectively (**Figure 3**). The median rwPFS was 6.2 months (95% CI, 5.5–7.1), with overall estimated rwPFS of 29.5% at 12 months and 14.3% at 24 months. The 12-month rwPFS rates were 39.1%, 21.1%, 24.5%, and 36.1% in PD-L1 ≥50%, 1–49%, <1%, and unknown cohorts, respectively (**Figure 4A**). Results were similar with censoring of clinician-defined pseudoprogression in the sensitivity analysis (overall median rwPFS, 6.4 months; 95% CI, 5.6–7.3; **Figure 4B**).

The rwRR was 39.3%; 22 patients (6%) had CR and 126 patients (33%) had PR (**Table 4**). (These 148 patients represented 50% of the 296 patients with recorded rwR, as 81 patients had no evaluable rwR assessment.) For these 148 patients, the median time to response was 2.3 months (range, 1.1–29.6 months), and the Kaplan-Meier estimate of rwDOR was 13.1 months (95% CI, 10.5–16.8; **Table 4**).

**Table 4:** Real-world tumor response.

		PD-L1 expression level			
Outcome	All patients N = 377	≥50% n = 105	1–49% n = 104	<1% n = 103	Unknown n = 65
Real-world tumor response, n	148	53	40	27	28
rwRR, % (95% CI)^a^	39.3 (34.3–44.4)	50.5 (40.5–60.4)	38.5 (29.1–48.5)	26.2 (18.0–35.8)	43.1 (30.8–56.0)
rwR category, n (%)^b^					
Complete response	22 (5.8)	13 (12.4)	2 (1.9)	5 (4.9)	2 (3.1)
Partial response	126 (33.4)	40 (38.1)	38 (36.5)	22 (21.4)	26 (40.0)
Stable disease	87 (23.1)	20 (19.0)	23 (22.1)	32 (31.1)	12 (18.5)
Progressive disease	61 (16.2)	12 (11.4)	19 (18.3)	22 (21.4)	8 (12.3)
No evaluable assessment, n (%)^c^	81 (21.5)	20 (19.0)	22 (21.2)	22 (21.4)	17 (26.2)
Pseudoprogression	3 (0.8)	0	2 (1.9)	1 (1.0)	0
Not documented/unknown	79 (21.0)	20 (19.0)	21 (20.2)	21 (20.4)	17 (26.2)
Months to response, median (range)	2.3 (1.1–29.6)	2.5 (1.1–29.6)	2.2 (1.1–24.8)	2.5 (1.1–20.3)	2.0 (1.2–25.8)
rwDOR, median (95% CI)^d^	13.1 (10.5–16.8)	16.8 (10.2–25.5)	10.4 (6.4–26.7)	11.6 (6.0–15.1)	16.7 (10.4–NA)
Range, mo	0.8+ to 34.7+	0.8+ to 34.7+	3.1+ to 31.5+	2.5+ to 29.6+	1.3+ to 32.0+

a The rwRR included patients with at least one CR or PR determination, followed by a subsequent assessment of CR, PR, or stable disease during the course of first-line therapy.

b For patients with multiple rwR assessments, the best response was used (CR>PR>stable disease>PD).

c Patients without an evaluable rwR assessment may be counted more than once.

d rwDOR was determined using Kaplan-Meier, with censoring of patients who initiated second-line therapy.

+, ongoing response; CR, complete response; PR, partial response; PD, progressive disease; rwDOR, real-world duration of response; rwR, real-world tumor response assessment.

## Discussion

This large retrospective study included 377 patients with *EGFR/ALK* wild-type, metastatic nonsquamous NSCLC treated with first-line pembrolizumab-combination therapy whom we were able to follow for a median of 31 months until data cutoff at the end of August 2020. Overall, the median pembrolizumab rwToT was 5.8 months, and the median pemetrexed rwToT was 2.7 months, similar to rwToT in our prior study (5.6 and 2.8 months, respectively (17)). On-treatment rates for pembrolizumab were 28.0% and 14.9% at 12 and 24 months, respectively. The Kaplan-Meier median OS was 17.2 months (95% CI, 13.6–19.9), with estimated 12- and 24-month survival rates of 59.4% and 39.2%, and median rwPFS was 6.2 months (95% CI, 5.5–7.1). These findings are in line with the findings of our prior study of stage IV nonsquamous NSCLC treated with first-line pembrolizumab-combination therapy (median OS of 16.5 months, with 12-month survival rate of 59.5%, and median rwPFS of 6.4 months) (17). At 12 months, survival rates by PD-L1 TPS status were 66.0%, 58.5%, 54.5%, and 58.3% for PD-L1 ≥50%, 1–49%, <1%, and unknown cohorts, respectively, also similar to our prior study (65.1%, 59.6%, 54.3%, and 58.4%, respectively).

The follow-up times in the present study resembled those for the KEYNOTE-189 clinical trial at the protocol-specified final analysis: namely, median of 31.2 months from first-line therapy initiation to data cutoff in the present study and 31.0 months from randomization to data cutoff in KEYNOTE-189; and median follow-up times to death/data cut-off were 18.9 and 18.8 months, respectively (11). However, the median pembrolizumab rwToT in the present study (5.8 months), and particularly the median pemetrexed rwToT (2.7 months), were both shorter than the median duration of therapy in the pembrolizumab plus pemetrexed-platinum group in the KEYNOTE-189 final analysis (7.2 months). Moreover, we observed that the real-world survival outcomes were somewhat inferior to those in KEYNOTE-189 (11). For example, median OS was 17.2 months in the present study and 22.2 months in KEYNOTE-189; 24-month survival rates were 39.2% and 45.7%, respectively; and median rwPFS/PFS was 6.2 months and 9.0 months, respectively (11).

Demographic characteristics of the real-world patients in this study were similar to those of clinical trial patients (median age 66 years vs. 65 years in the pembrolizumab-chemotherapy arm of KEYNOTE-189 and 40% vs. 38% women, respectively), and similar percentages of patients had ECOG PS of 1 (56% and 54% in KEYNOTE-189 (9)). However, we could not rule out whether the real-world patients may have had comorbidities that would have been cause for exclusion from KEYNOTE-189, for example, symptomatic central nervous system metastases, history of noninfectious pneumonitis requiring glucocorticoids, or active autoimmune disease (9). Moreover, we note that just 39.0% of patients continued to a subsequent line of systemic therapy, in contrast to 49.5% in the pembrolizumab-chemotherapy arm of KEYNOYE-189 (11). Two studies in the Netherlands comparing real-world with clinical trial outcomes for patients with stage IV NSCLC, one in the preimmunotherapy era and one in 2015–2018, also found that fewer real-world patients proceeded to a subsequent line of therapy (30, 31). In clinical settings, physicians may have a lower threshold to stop or change therapy in response to minor imaging changes, and perhaps associated clinical deterioration.

In a recent US observational study of patients with nonsquamous advanced NSCLC who received first-line immunotherapy-chemotherapy and had an ECOG PS 0–1, the reported median OS was 14.2 months, somewhat shorter than in the present study (17.2 months), and estimated 12-month and 24-month OS rates were 54.8% and 36.8% (vs. 59.4% and 39.2% in the present study) (13). However, that study, which also drew on the Flatiron Health database, included patients with unknown *EGFR/ALK* mutation status (~18%) and had median follow-up of only 6.5 months. The Kaplan-Meier median duration of first-line immunotherapy-chemotherapy (5.6 months) was similar to that in the present study (median rwToT, 5.8 months). The authors identified older age, ECOG PS >1, and presence of brain metastases as being associated with poorer survival outcomes, and PD-L1 expression of ≥50% as being associated with longer median OS (vs. low expression) (13). For an older US population (ages 66–89) of 1495 patients with advanced NSCLC, Kehl et al. (19) reported median OS of 12.9 months (95% CI, 11.8–14.0) with pembrolizumab-pemetrexed-platinum-based chemotherapy. Their study used Medicare fee-for-service claims, which did not include tumor histopathology, status of actionable genomic alterations, PD-L1 expression level, or ECOG PS.

Other observational studies of first-line ICI-chemotherapy for metastatic NSCLC conducted outside the US were also not restricted to nonsquamous NSCLC. Dudnik et al. (32) reported median OS of 20.4 months (95% CI, 10.8-not reached) for 53 patients, most with good performance status (85% ECOG PS 0–1), with *EGFR/ALK/ROS1*-wild-type advanced NSCLC, PD-L1 ≥50% who received first-line ICI plus platinum-based chemotherapy at Israeli cancer centers. In another retrospective non-US study (at the Shanghai Chest Hospital), median OS was not reached for 115 patients with advanced NSCLC (both nonsquamous and squamous), PD-L1 ≥50%, and no *EGFR* or *ALK* genomic alterations who received first-line pembrolizumab-chemotherapy (33). In the latter study, all patients had an ECOG PS of 0 or 1.

In KEYNOTE-189, the benefits of pembrolizumab-combination therapy, as compared with placebo-combination therapy, were observed irrespective of PD-L1 expression. We found, similar to Waterhouse et al. (13), that survival was longer for patients with nonsquamous NSCLC with PD-L1 expression ≥50%, among whom median OS was 20.6 months (95% CI, 16.2–26.8), while for those with PD-L1 <1%, median OS was 13.2 months (95% CI, 10.9–19.9). In addition, there were other differences between the PD-L1 ≥50% vs. PD-L1 <1% cohorts in the present study, namely, the median rwPFS was longer for the PD-L1 ≥50% cohort (9.3 months vs. 5.0 months for PD-L1 <1%), and at 24 months, more patients remained on pembrolizumab therapy (18.4% vs. 9.4%, respectively), while therapy-related adverse events resulting in pembrolizumab discontinuation were slightly greater for the PD-L1 ≥50% cohort (21.1% vs. 14.8%, respectively). However, we believe these results should be interpreted with caution for two reasons: (i) this study was not designed for PD-L1 cohort comparisons, and (ii) the patient numbers in each PD-L1 cohort were relatively small (n≈100).

Peters et al. (15) reported median OS of 21.0 months and rwPFS of 10.8 months for 169 patients with metastatic nonsquamous NSCLC, PD-L1 ≥50%, ECOG PS 0–1 who initiated first-line ICI-chemotherapy from 24 October 2016 through February 2019, similar to our findings and indeed with possible overlap of individual patients, as their study used the Flatiron Health database and covered the span of first-line therapy initiation in our study (11 May 2017 through 31 January 2019).

A strength of our study is the manual chart review to assess two endpoints that have been studied and correlated with OS: (i) a clinician-anchored approach supported by radiology report data to determine rwP (24) and (ii) a real-world response variable (rwR) that also showed good correlation with clinical trial overall response rates (28). In addition, we were able to use the Flatiron Health database, regarded as a well-curated database, and to include a patient population well-characterized with regard to actionable genomic alterations and PD-L1 status, the latter missing for just 17% of patients. Median follow-up of 31 months from pembrolizumab-combination initiation to data cutoff was longer than in our prior study and other recent observational studies of ICI therapy in the US that reported follow-up length (13–17).

### Limitations

Most patients included in this study were treated in the community oncology setting; therefore, results may not be generalizable to patients treated at academic centers, or to those treated outside the Flatiron Health network. Moreover, because *EGFR/ALK* status and ECOG PS were not consistently documented in the Flatiron Health database, otherwise potentially eligible patients were excluded, raising the potential for selection bias. The database was missing information for clinically important variables, such as PD-L1 expression (17% missing), *KRAS* mutation status (31% missing), and whether brain metastases were pretreated or symptomatic, although observational studies suggest that the survival benefit from pembrolizumab-based therapy may not be inferior for patients with brain metastases (16). In addition, information to determine rwR was not available for 22% of patients.

Continuing study is needed to compare real-world outcomes for patients with and without brain metastases and to assess whether *KRAS* mutation status has an impact on the survival of patients with metastatic NSCLC who are treated in first-line with ICI-chemotherapy.

## Conclusions

The findings of this retrospective study of patients with *EGFR/ALK*-wild-type, metastatic nonsquamous NSCLC treated with first-line pembrolizumab-combination therapy serve to illustrate outcomes achieved in the real-world setting of community oncology practice in the US for patients with good performance status. The median OS of 17.2 months, with estimated 12- and 24-month survival rates of 59.4% and 39.2%, suggest benefits of first-line pembrolizumab-combination therapy approaching those recorded in the clinical trial setting. Moreover, these findings update and support the findings of our prior study (17) with a larger patient population followed for a longer period of time.

## Data availability statement

The data that support the findings of this study have been originated by Flatiron Health, Inc. These deidentified data may be made available upon request and are subject to a license agreement with Flatiron Health; interested researchers should contact DataAccess@flatiron.com to determine licensing terms. Requests to access the datasets should be directed to DataAccess@flatiron.com.

## Ethics statement

The studies involving human participants were reviewed and approved by WCG Institutional Review Board. Written informed consent for participation was not required for this study in accordance with the national legislation and the institutional requirements.

## Author contributions

Conception and design of the study: SVL, XH, YL, BZ, TB, VV. Data analysis: YL. Interpretation of findings, critical review, and revision of the manuscript: SVL, XH, YL, BZ, TB, VV. All authors contributed to the article and approved the submitted version.

## Funding

This work was supported by Merck Sharp & Dohme LLC, a subsidiary of Merck & Co., Inc., Rahway, NJ, USA. The funder of the study participated in development of the study design and funded the analysis of the data.

## Acknowledgments

We gratefully acknowledge Shikha Surati for administrative support and Benjamin L. Koch and Mary Anne Rutkowski for programming support (all of Merck & Co., Inc., Rahway, NJ, USA). Medical writing and editorial assistance were provided by Elizabeth V. Hillyer, DVM (freelance). This assistance was funded by Merck Sharp & Dohme LLC, a subsidiary of Merck & Co., Inc., Rahway, NJ, USA.

## Conflict of interest

SVL reports serving in an advisory and/or consultant role for Amgen, AstraZeneca, Bayer, Beigene, Blueprint, Boehringer-Ingelheim, Bristol Myers Squibb, Catalyst, Daiichi Sankyo, Eisai, Elevation Oncology, Genentech, Gilead, Guardant Health, Janssen, Jazz Pharmaceuticals, Lilly, MSD, Novartis, Regeneron, Sanofi, Takeda, and Turning Point Therapeutics; receiving research funding for his institution from Alkermes, Bayer, Blueprint, Bristol-Myers Squibb, Elevation Oncology, Genentech, Gilead, Lilly, MSD, Merus, Nuvalent, Pfizer, Rain Therapeutics, RAPT, and Turning Point Therapeutics. XH, YL, BZ, and TB are full-time employees of Merck Sharp & Dohme LLC, a subsidiary of Merck & Co., Inc., Rahway, NJ, USA and own stock of Merck & Co., Inc., Rahway, NJ, USA. VV reports serving in an advisory and/or consultant role for MSD, Bristol-Myers Squibb, AstraZeneca, Novartis, Amgen, Bayer, and Foundation Medicine.

## Publisher’s note

All claims expressed in this article are solely those of the authors and do not necessarily represent those of their affiliated organizations, or those of the publisher, the editors and the reviewers. Any product that may be evaluated in this article, or claim that may be made by its manufacturer, is not guaranteed or endorsed by the publisher.
