# Supplementary material for: Pembrolizumab-combination therapy for previously untreated metastatic nonsquamous NSCLC: Real-world outcomes at US oncology practices
Source: Front Oncol. 2022 Oct 17;12:999343. doi: 10.3389/fonc.2022.999343 (PMC9618586; doi:10.3389/fonc.2022.999343)
Supplement: Supplementary file 1 [file DataSheet_1.docx]

**Supplementary TABLE 1 |** Subsequent systemic anticancer therapy lines and regimens

|  | **All patients**  **N = 377** | **PD-L1 expression level** | | | |
| --- | --- | --- | --- | --- | --- |
|  |  | **≥50%**  **n = 105** | **1–49%**  **n = 104** | **<1%**  **n = 103** | **Unknown**  **n = 65** |
| **Second-line regimen, n (%)** | **147 (39.0)** | **42 (40.0)** | **45 (43.3)** | **43 (41.7)** | **17 (26.2)** |
| ***Anti-PD-1/PD-L1-based therapy*** | **35 (23.8)** | **8 (19.0)** | **16 (35.6)** | **5 (11.6)** | **6 (35.3)** |
| Pembrolizumab | 11 (31.4) | 4 (50.0) | 5 (31.2) | 1 (20.0) | 1 (16.7) |
| Pembrolizumab, pemetrexed | 5 (14.3) | 0 | 3 (18.8) | 0 | 2 (33.3) |
| Nivolumab | 3 (8.6) | 0 | 1 (6.2) | 2 (40.0) | 0 |
| Atezolizumab, bevacizumab, carboplatin, paclitaxel | 2 (5.7) | 1 (12.5) | 1 (6.2) | 0 | 0 |
| Carboplatin, pembrolizumab, pemetrexed | 3 (8.6) | 1 (12.5) | 1 (6.2) | 1 (20.0) | 0 |
| Carboplatin, paclitaxel, pembrolizumab | 3 (8.6) | 1 (12.5) | 1 (6.2) | 1 (20.0) | 0 |
| Atezolizumab | 1 (2.9) | 0 | 1 (6.2) | 0 | 0 |
| Bevacizumab-awwb, pembrolizumab | 1 (2.9) | 1 (12.5) | 0 | 0 | 0 |
| Ipilimumab, nivolumab | 1 (2.9) | 0 | 0 | 0 | 1 (16.7) |
| Bevacizumab, carboplatin, paclitaxel, pembrolizumab, pemetrexed | 0 | 0 | 0 | 0 | 0 |
| Carboplatin, nivolumab, paclitaxel | 0 | 0 | 0 | 0 | 0 |
| Bevacizumab, pembrolizumab, pemetrexed | 1 (2.9) | 0 | 1 (6.2) | 0 | 0 |
| Atezolizumab, bevacizumab, carboplatin, paclitaxel protein-bound | 1 (2.9) | 0 | 0 | 0 | 1 (16.7) |
| Clinical study drug, pembrolizumab, pemetrexed | 1 (2.9) | 0 | 0 | 0 | 1 (16.7) |
| Atezolizumab, bevacizumab-awwb, carboplatin, paclitaxel | 1 (2.9) | 0 | 1 (6.2) | 0 | 0 |
| Gemcitabine, pembrolizumab | 0 | 0 | 0 | 0 | 0 |
| Bevacizumab-awwb, carboplatin, paclitaxel, pembrolizumab | 0 | 0 | 0 | 0 | 0 |
| Carboplatin, paclitaxel protein-bound, pembrolizumab | 0 | 0 | 0 | 0 | 0 |
| Paclitaxel, pembrolizumab | 1 (2.9) | 0 | 1 (6.2) | 0 | 0 |
| Afatinib, carboplatin, pembrolizumab, pemetrexed | 0 | 0 | 0 | 0 | 0 |
| ***Anti-VEGF-based therapy*** | **43 (29.3)** | **13 (31.0)** | **6 (13.3)** | **20 (46.5)** | **4 (23.5)** |
| Docetaxel, ramucirumab | 30 (69.8) | 9 (69.2) | 4 (66.7) | 16 (80.0) | 1 (25.0) |
| Bevacizumab, carboplatin, paclitaxel | 2 (4.7) | 1 (7.7) | 0 | 0 | 1 (25.0) |
| Bevacizumab, carboplatin, pemetrexed | 3 (7.0) | 1 (7.7) | 0 | 1 (5.0) | 1 (25.0) |
| Bevacizumab, carboplatin, paclitaxel protein-bound | 1 (2.3) | 0 | 0 | 1 (5.0) | 0 |
| Bevacizumab, carboplatin, gemcitabine | 2 (4.7) | 1 (7.7) | 1 (16.7) | 0 | 0 |
| Bevacizumab, docetaxel, ramucirumab | 2 (4.7) | 1 (7.7) | 0 | 0 | 1 (25.0) |
| Bevacizumab, paclitaxel protein-bound | 1 (2.3) | 0 | 1 (16.7) | 0 | 0 |
| Ramucirumab | 0 | 0 | 0 | 0 | 0 |
| Bevacizumab, carboplatin, docetaxel | 0 | 0 | 0 | 0 | 0 |
| Bevacizumab, docetaxel | 0 | 0 | 0 | 0 | 0 |
| Bevacizumab | 1 (2.3) | 0 | 0 | 1 (5.0) | 0 |
| Bevacizumab, gemcitabine | 1 (2.3) | 0 | 0 | 1 (5.0) | 0 |
| ***Platinum-based chemotherapy combination*** | **16 (10.9)** | **5 (11.9)** | **6 (13.3)** | **3 (7.0)** | **2 (11.8)** |
| Carboplatin, paclitaxel | 10 (62.5) | 3 (60.0) | 4 (66.7) | 1 (33.3) | 2 (100) |
| Carboplatin, pemetrexed | 3 (18.8) | 2 (40.0) | 1 (16.7) | 0 | 0 |
| Carboplatin, paclitaxel protein-bound | 1 (6.2) | 0 | 0 | 1 (33.3) | 0 |
| Carboplatin, gemcitabine | 1 (6.2) | 0 | 0 | 1 (33.3) | 0 |
| Carboplatin, paclitaxel protein-bound, pemetrexed | 0 | 0 | 0 | 0 | 0 |
| Carboplatin, docetaxel | 1 (6.2) | 0 | 1 (16.7) | 0 | 0 |
| ***Nonplatinum-based chemo combination*** | **7 (4.8)** | **2 (4.8)** | **1 (2.2)** | **4 (9.3)** | **0** |
| Paclitaxel protein-bound | 4 (57.1) | 1 (50.0) | 0 | 3 (75.0) | 0 |
| Gemcitabine, vinorelbine | 2 (28.6) | 0 | 1 (100) | 1 (25.0) | 0 |
| Docetaxel, gemcitabine | 1 (14.3) | 1 (50.0) | 0 | 0 | 0 |
| ***Single agent chemotherapy*** | **28 (19.0)** | **8 (19.0)** | **9 (20.0)** | **8 (18.6)** | **3 (17.6)** |
| Docetaxel | 15 (53.6) | 2 (25.0) | 4 (44.4) | 7 (87.5) | 2 (66.7) |
| Gemcitabine | 8 (28.6) | 4 (50.0) | 3 (33.3) | 1 (12.5) | 0 |
| Paclitaxel | 5 (17.9) | 2 (25.0) | 2 (22.2) | 0 | 1 (33.3) |
| ***Other therapy*** | **18 (12.2)** | **6 (14.3)** | **7 (15.6)** | **3 (7.0)** | **2 (11.8)** |
| Clinical study drug | 7 (38.9) | 3 (50.0) | 2 (28.6) | 1 (33.3) | 1 (50.0) |
| Crizotinib | 3 (16.7) | 1 (16.7) | 1 (14.3) | 1 (33.3) | 0 |
| Rucaparib | 2 (11.1) | 1 (16.7) | 0 | 1 (33.3) | 0 |
| Ado-trastuzumab emtansine | 2 (11.1) | 1 (16.7) | 1 (14.3) | 0 | 0 |
| Durvalumab | 1 (5.6) | 0 | 1 (14.3) | 0 | 0 |
| Midostaurin | 0 | 0 | 0 | 0 | 0 |
| Binimetinib, dabrafenib, encorafenib, trametinib | 1 (5.6) | 0 | 0 | 0 | 1 (50.0) |
| Afatinib, docetaxel, ramucirumab | 1 (5.6) | 0 | 1 (14.3) | 0 | 0 |
| Afatinib | 0 | 0 | 0 | 0 | 0 |
| Dabrafenib, trametinib | 1 (5.6) | 0 | 1 (14.3) | 0 | 0 |
| **Third-line regimen, n (%)** | **65 (17.2)** | **16 (15.2)** | **20 (19.2)** | **21 (20.4)** | **8 (12.3)** |
| ***Anti-PD-1/PD-L1-based therapy*** | **10 (15.4)** | **4 (25.0)** | **3 (15.0)** | **2 (9.5)** | **1 (12.5)** |
| Pembrolizumab | 4 (40.0) | 1 (25.0) | 3 (100) | 0 | 0 |
| Nivolumab | 3 (30.0) | 1 (25.0) | 0 | 2 (100) | 0 |
| Carboplatin, pembrolizumab, pemetrexed | 1 (10.0) | 1 (25.0) | 0 | 0 | 0 |
| Carboplatin, paclitaxel, pembrolizumab | 0 | 0 | 0 | 0 | 0 |
| Pembrolizumab, pemetrexed | 0 | 0 | 0 | 0 | 0 |
| Atezolizumab, bevacizumab-awwb, carboplatin, paclitaxel protein-bound | 1 (10.0) | 0 | 0 | 0 | 1 (100) |
| Bevacizumab-bvzr, pembrolizumab | 1 (10.0) | 1 (25.0) | 0 | 0 | 0 |
| ***Anti-VEGF-based therapy*** | **21 (32.3)** | **3 (18.8)** | **9 (45.0)** | **6 (28.6)** | **3 (37.5)** |
| Docetaxel, ramucirumab | 10 (47.6) | 2 (66.7) | 5 (55.6) | 3 (50.0) | 0 |
| Bevacizumab, carboplatin, paclitaxel | 3 (14.3) | 0 | 1 (11.1) | 0 | 2 (66.7) |
| Bevacizumab-awwb, carboplatin, pemetrexed | 2 (9.5) | 1 (33.3) | 1 (11.1) | 0 | 0 |
| Bevacizumab, gemcitabine | 0 | 0 | 0 | 0 | 0 |
| Docetaxel, paclitaxel, ramucirumab | 1 (4.8) | 0 | 1 (11.1) | 0 | 0 |
| Bevacizumab, dabrafenib, paclitaxel protein-bound, trametinib | 1 (4.8) | 0 | 0 | 1 (16.7) | 0 |
| Bevacizumab, pemetrexed | 1 (4.8) | 0 | 0 | 0 | 1 (33.3) |
| Bevacizumab, bevacizumab-awwb | 1 (4.8) | 0 | 0 | 1 (16.7) | 0 |
| Bevacizumab-awwb, docetaxel, gemcitabine, paclitaxel | 0 | 0 | 0 | 0 | 0 |
| Docetaxel, gemcitabine, ramucirumab | 1 (4.8) | 0 | 1 (11.1) | 0 | 0 |
| Bevacizumab-awwb | 1 (4.8) | 0 | 0 | 1 (16.7) | 0 |
| ***Platinum-based chemotherapy combination*** | **3 (4.6)** | **0** | **1 (5.0)** | **1 (4.8)** | **1 (12.5)** |
| Carboplatin, gemcitabine | 1 (33.3) | 0 | 0 | 0 | 1 (100) |
| Cisplatin, gemcitabine | 1 (33.3) | 0 | 1 (100) | 0 | 0 |
| Carboplatin, paclitaxel protein-bound | 1 (33.3) | 0 | 0 | 1 (100) | 0 |
| ***Nonplatinum-based chemo combination*** | **4 (6.2)** | **0** | **0** | **3 (14.3)** | **1 (12.5)** |
| Gemcitabine, vinorelbine | 2 (50.0) | 0 | 0 | 1 (33.3) | 1 (100) |
| Paclitaxel protein-bound | 1 (25.0) | 0 | 0 | 1 (33.3) | 0 |
| Gemcitabine, paclitaxel protein-bound | 1 (25.0) | 0 | 0 | 1 (33.3) | 0 |
| Durvalumab, vinorelbine | 0 | 0 | 0 | 0 | 0 |
| ***Single agent chemotherapy*** | **23 (35.4)** | **6 (37.5)** | **7 (35.0)** | **8 (38.1)** | **2 (25.0)** |
| Gemcitabine | 16 (69.6) | 4 (66.7) | 4 (57.1) | 6 (75.0) | 2 (100) |
| Docetaxel | 3 (13.0) | 0 | 2 (28.6) | 1 (12.5) | 0 |
| Vinorelbine | 3 (13.0) | 1 (16.7) | 1 (14.3) | 1 (12.5) | 0 |
| Pemetrexed | 1 (4.3) | 1 (16.7) | 0 | 0 | 0 |
| ***Other therapy*** | **4 (6.2)** | **3 (18.8)** | **0** | **1 (4.8)** | **0** |
| Clinical study drug | 1 (25.0) | 0 | 0 | 1 (100) | 0 |
| Ado-trastuzumab emtansine | 1 (25.0) | 1 (33.3) | 0 | 0 | 0 |
| Olaparib | 0 | 0 | 0 | 0 | 0 |
| Crizotinib | 1 (25.0) | 1 (33.3) | 0 | 0 | 0 |
| Selpercatinib | 1 (25.0) | 1 (33.3) | 0 | 0 | 0 |
| **Fourth-line regimen, n (%)** | **26 (6.9)** | **8 (7.6)** | **8 (7.7)** | **9 (8.7)** | **1 (1.5)** |
| ***Anti-PD-1/PD-L1-based therapy*** | **2 (7.7)** | **1 (12.5)** | **0** | **1 (11.1)** | **0** |
| Atezolizumab | 0 | 0 | 0 | 0 | 0 |
| Ipilimumab, nivolumab | 1 (50.0) | 1 (100) | 0 | 0 | 0 |
| Pembrolizumab, vinorelbine | 0 | 0 | 0 | 0 | 0 |
| Nivolumab | 1 (50.0) | 0 | 0 | 1 (100) | 0 |
| ***Anti-VEGF-based therapy*** | **5 (19.2)** | **1 (12.5)** | **2 (25.0)** | **1 (11.1)** | **1 (100)** |
| Bevacizumab, pemetrexed | 1 (20.0) | 1 (100) | 0 | 0 | 0 |
| Bevacizumab, bevacizumab-awwb, carboplatin, paclitaxel protein-bound | 1 (20.0) | 0 | 1 (50.0) | 0 | 0 |
| Bevacizumab, paclitaxel protein-bound | 1 (20.0) | 0 | 0 | 0 | 1 (100) |
| Bevacizumab-awwb, carboplatin, pemetrexed | 1 (20.0) | 0 | 1 (50.0) | 0 | 0 |
| Bevacizumab-bvzr | 1 (20.0) | 0 | 0 | 1 (100) | 0 |
| ***Platinum-based chemotherapy combination*** | **1 (3.8)** | **0** | **0** | **1 (11.1)** | **0** |
| Carboplatin, paclitaxel | 0 | 0 | 0 | 0 | 0 |
| Carboplatin, gemcitabine | 1 (100) | 0 | 0 | 1 (100) | 0 |
| ***Nonplatinum-based chemo combination*** | **4 (15.4)** | **2 (25.0)** | **1 (12.5)** | **1 (11.1)** | **0** |
| Paclitaxel protein-bound | 2 (50.0) | 1 (50.0) | 0 | 1 (100) | 0 |
| Gemcitabine, olaparib | 1 (25.0) | 0 | 1 (100) | 0 | 0 |
| Gemcitabine, vinorelbine | 1 (25.0) | 1 (50.0) | 0 | 0 | 0 |
| ***Single agent chemotherapy*** | **11 (42.3)** | **3 (37.5)** | **4 (50.0)** | **4 (44.4)** | **0** |
| Gemcitabine | 4 (36.4) | 1 (33.3) | 2 (50.0) | 1 (25.0) | 0 |
| Vinorelbine | 4 (36.4) | 1 (33.3) | 1 (25.0) | 2 (50.0) | 0 |
| Docetaxel | 2 (18.2) | 1 (33.3) | 1 (25.0) | 0 | 0 |
| Pemetrexed | 0 | 0 | 0 | 0 | 0 |
| Paclitaxel | 1 (9.1) | 0 | 0 | 1 (25.0) | 0 |
| ***Other therapy*** | **3 (11.5)** | **1 (12.5)** | **1 (12.5)** | **1 (11.1)** | **0** |
| Selpercatinib | 1 (33.3) | 0 | 0 | 1 (100) | 0 |
| Capmatinib, crizotinib | 1 (33.3) | 1 (100) | 0 | 0 | 0 |
| Dabrafenib, trametinib | 1 (33.3) | 0 | 1 (100) | 0 | 0 |
| **Fifth-line regimen, n (%)** | **8 (2.1)** | **1 (1.0)** | **2 (1.9)** | **4 (3.9)** | **1 (1.5)** |
| ***Anti-VEGF-based therapy*** | **2 (25.0)** | **0** | **1 (50.0)** | **0** | **1 (100)** |
| Docetaxel, ramucirumab | 1 (50.0) | 0 | 1 (100) | 0 | 0 |
| Bevacizumab-awwb | 1 (50.0) | 0 | 0 | 0 | 1 (100) |
| ***Platinum-based chemotherapy combination*** | **1 (12.5)** | **0** | **1 (50.0)** | **0** | **0** |
| Carboplatin, etoposide | 1 (100) | 0 | 1 (100) | 0 | 0 |
| ***Single agent chemotherapy*** | **4 (50.0)** | **1 (100)** | **0** | **3 (75.0)** | **0** |
| Vinorelbine | 2 (50.0) | 1 (100) | 0 | 1 (33.3) | 0 |
| Paclitaxel | 0 | 0 | 0 | 0 | 0 |
| Gemcitabine | 1 (25.0) | 0 | 0 | 1 (33.3) | 0 |
| Pemetrexed | 1 (25.0) | 0 | 0 | 1 (33.3) | 0 |
| ***Other therapy*** | **1 (12.5)** | **0** | **0** | **1 (25.0)** | **0** |
| Erlotinib | 1 (100) | 0 | 0 | 1 (100) | 0 |
| **Sixth-line regimen, n (%)** | **2 (0.5)** | **0** | **2 (1.9)** | **0** | **0** |
| ***Anti-PD-1/PD-L1-based therapy*** | **1 (50.0)** | **0** | **1 (50.0)** | **0** | **0** |
| Pembrolizumab | 1 (100) | 0 | 1 (100) | 0 | 0 |
| ***Single agent chemotherapy*** | **1 (50.0)** | **0** | **1 (50.0)** | **0** | **0** |
| Gemcitabine | 1 (100) | 0 | 1 (100) | 0 | 0 |

Drug regimens are shown as percentage of the relevant treatment line. Percentages may not total 100 because of rounding. For each line of therapy, mutually exclusive regimen classes were assigned in hierarchical order as follows: anti-PD-1/PD-L1-based therapy > anti-VEGF-based therapy > platinum-based chemotherapy combinations > nonplatinum-based chemotherapy combinations > single agent chemotherapy > other therapy.

Chemo, chemotherapy; PD-1, programmed death 1; PD-L1, PD-ligand 1; VEGF, vascular endothelial growth factor.

**Supplementary TABLE 2 |** rwToT of immune checkpoint inhibitors (ICI) of PD-1/PD-L1 administered in second- and third-line

| **Outcome** | **All patients** | **PD-L1 expression level** | | | |
| --- | --- | --- | --- | --- | --- |
|  |  | **≥50%** | **1–49%** | **<1%** | **Unknown** |
| **ICI in 2L, N** | **36** | **8** | **17** | **5** | **6** |
| Discontinued, n (%) | 24 (66.7) | 3 (37.5) | 14 (82.4) | 4 (80.0) | 3 (50.0) |
| rwToT, median (95% CI), mo | 2.8 (1.5–5.1) | 3.5 (1.4–NA) | 3.0 (1.0–5.3) | 2.8 (0.5–NA) | 2.4 (0–NA) |
| On-treatment |  |  |  |  |  |
| At 6 months, % (95% CI) | 27.4 (12.8–44.3) | 40.0 (5.2–75.3) | 22.6 (5.9–45.7) | 20.0 (0.8–58.2) | 41.7 (5.6–76.7) |
| At 12 months, % (95% CI) | 27.4 (12.8–44.3) | 0 (0–0) | 22.6 (5.9–45.7) | 0 (0–0) | 41.7 (5.6–76.7) |
| At 24 months, % (95% CI) | 13.7 (1.4–39.5) | 0 (0–0) | 11.3 (0.9–36.5) | 0 (0–0) | 0 (0–0) |
| **ICI in 3L, N** | **10** | **4** | **3** | **2** | **1** |
| Discontinued, n (%) | 5 (50.0) | 3 (75.0) | 1 (33.3) | 1 (50.0) | 1 (100) |
| rwToT, median (95% CI), mo | 4.3 (1.4–NA) | 1.9 (1.4–NA) | NA (1.6–NA) | 4.3 (NA–NA) | NA (NA–NA) |
| On-treatment |  |  |  |  |  |
| At 6 months, % (95% CI) | 46.9 (12–76.3) | 33.3 (0.9–77.4) | 66.7 (5.4–94.5) | 0 (0–0) | 100 (0–0) |
| At 12 months, % (95% CI) | 0 (0–0) | 0 (0–0) | 66.7 (5.4–94.5) | 0 (0–0) | 100 (0–0) |

2L, second-line; 3L, third-line.

**Supplementary TABLE 3 |** Reasons for first-line pembrolizumab discontinuation by patients administered an immune checkpoint inhibitor of PD-1/PD-L1 in second- and/or third-line

|  |  | **PD-L1 expression level** | | | |
| --- | --- | --- | --- | --- | --- |
|  | **All patients**  **N = 43** | **≥50%**  **n = 12** | **1–49%**  **n = 18** | **<1%**  **n = 7** | **Unknown**  **n = 6** |
| Discontinued, n (%) | 34 (79.1) | 9 (75.0) | 14 (77.8) | 6 (85.7) | 5 (83.3) |
| Reasons for discontinuation, n (%)^a^ |  |  |  |  |  |
| Progression | 21 (61.8) | 6 (66.7) | 12 (85.7) | 2 (33.3) | 1 (20.0) |
| Adverse events related to therapy | 7 (20.6) | 1 (11.1) | 1 (7.1) | 3 (50.0) | 2 (40.0) |
| Disease-related symptoms not due to therapy | 1 (2.9) | 1 (11.1) | 0 | 0 | 0 |
| Patient request | 3 (8.8) | 1 (11.1) | 1 (7.1) | 0 | 1 (20.0) |
| Completed treatment | 0 | 0 | 0 | 0 | 0 |
| No evidence of disease | 0 | 0 | 0 | 0 | 0 |
| Financial | 0 | 0 | 0 | 0 | 0 |
| Other^b^ | 2 (5.9) | 0 | 0 | 1 (16.7) | 1 (20.0) |
| Unknown | 0 | 0 | 0 | 0 | 0 |

Data are n (%) unless otherwise noted. Percentages may not add up to 100 because of rounding.

^a^ Patients could have more than one reason for discontinuation.

^b^ For patients with ongoing treatment until the time of death, the reason recorded was “Other” to comply with data deidentification requirements.
